# “A Blessing and a Curse”: The Impact of Sociopolitical Events and National Discourse Surrounding Sexual Violence on College Campuses

**DOI:** 10.1007/s10896-024-00705-z

**Published:** 2024-07-09

**Authors:** Danielle M. Davidov, Caterina DeFazio, Desireé N. Williford, Emily R. Clear, Heather M. Bush, Ann L. Coker

**Affiliations:** 1 School of Public Health, West Virginia University, Morgantown, WV USA; 2 Cincinnati Children’s Hospital Medical Center, Cincinnati, OH USA; 3 College of Public Health, University of Kentucky, Lexington, KY USA; 4 College of Medicine, Endowed Chair in the Center for Research On Violence Against Women, University of Kentucky, Lexington, KY USA

**Keywords:** Sexual assault, Bystander, #MeToo, Title IX, Qualitative, Violence prevention, University

## Abstract

**Purpose:**

Examining the influence of policy change and socio-political factors is paramount for contextualizing and addressing sexual violence. The purpose of this paper is to provide findings from a secondary qualitative analysis of the impacts of national and local high-profile events on the dialogue and actions surrounding violence prevention and response on college campuses.

**Methods:**

Data from mcBEE, a Centers for Disease Control and Prevention-funded project exploring the adoption and implementation of violence prevention programming on multiple college campuses in the United States were used for this analysis. Data include responses from 60-min telephone interviews with key informants (i.e., campus personnel in administrative roles or connected to violence prevention programming efforts) between 2017 and 2019.

**Results:**

Interviews (*n* = 68) revealed that high-profile events (i.e., Title IX legislation, news coverage of sexual assault cases, and the #MeToo and It’s On Us movements) increased dialogue about violence prevention and response on some campuses, while others experienced activism and advocacy surrounding sexual assault, including greater accountability and response from campus leadership. Some participants connected national political discourse (i.e., the 2016 presidential election, Brett Kavanaugh nomination to the U.S. Supreme Court) to increases in violence perpetration and decreased reporting and help-seeking behaviors after sexual assault.

**Conclusions:**

Sociopolitical events and shifts in national dialogue surrounding violence affect perceptions and behaviors among students, faculty, staff, and overall college campus communities. Identifying potential impacts of national events can inform future prevention and response efforts and mobilize campus communities toward meaningful change.

College-based sexual assault is a major public health problem, with students on college campuses reporting a wide range of victimization experiences. While estimates vary by definition and methodology, research has consistently documented that between 20 – 25% of women and 4 – 12% of men will be assaulted during their time in college (Banyard et al., 2007; Cantor et al., 2015; Cantor et al., 2020; Conley et al., 2017; Fedina et al., 2018; Krebs et al., 2007, 2009, 2016; Muehlenhard et al., 2017). Sexual minority and gender non-conforming college students, such as those who identify as trans or genderqueer, are often underrepresented in research, yet available data show they experience sexual violence victimization at rates equal to or higher than heterosexual women and nearly twice as high as their non-sexual minority and cisgender college-attending peers (Cantor et al., 2015, 2020; Edwards et al., 2015; Ford & Soto-Marquez, 2016). In most cases, the survivors know their attacker—either as an acquaintance, friend, or dating partner—and most assaults go unreported (Cantor et al., 2015, 2020; Fisher et al., 2000; Koss, 2011; Krebs et al., 2007).

For college students, sexual assault is associated with a host of long- and short-term negative socioeconomic and health-related consequences. A substantial body of research has demonstrated that sexual assault results in worse class attendance and grades as well as lower graduation rates (Jordan et al., 2014; Kaukinen, 2014; Mengo & Black, 2016; Molstad et al., 2023; Potter et al., 2018); these outcomes can impact lifelong earning potential and future employment. Further, aside from chronic and acute physical health consequences, mental health complications such as insomnia, depression, anxiety, and post-traumatic stress disorder are common among survivors of sexual assault (Carey et al., 2018; Dworkin, 2020; Rothman et al., 2021; Ullman & Brecklin, 2003).

In response to the harmful consequences of sexual assault among college populations, federal agencies took steps to enact legislation (e.g., Title IX, the Clery Act of 1990, the 1994 Violence Against Women Act) focused on violence prevention and response in higher education. Additionally, the 2013 Campus SaVE Act, which amends the Clery Act, mandated that colleges receiving federal funding provide all students with “primary prevention and awareness programs” related to sexual misconduct and assault. Within this mandate is language specific to providing students with “safe and positive options for bystander intervention” (American Council of Education, 2014).

Most U.S. colleges were tasked with adopting and implementing new violence prevention initiatives or modifying existing interventions to include bystander elements because of Campus SaVE. Thus, an opportunity exists to explore its impact by learning how schools have responded to bystander education requirements and evaluating the short- and long-term impacts of this programming on college student rates of sexual violence. At the same time, is it critical to examine how policy change and other societal forces and events may contribute to campus-decision-making or action surrounding program adoption and implementation.

## Sociopolitical Contexts of Sexual Violence

Societal attitudes and norms, often shaped by political and social factors, play a significant role in perpetuating or challenging the culture of silence around sexual assault and misconduct. Since the early 2010s, hashtag feminist movements and “digital activism” to call out instances of sexism and sexual violence have emerged (Gjika & Marganski, 2020; Rodino-Colocino, 2014). For example, the #MeToo and #TimesUp campaigns and increased media attention on high-profile (e.g., celebrity, athlete) reports of sexual and dating violence have undoubtedly shifted how sexual misconduct is viewed and discussed publicly (Kilimnik et al., 2024; Lokot, 2018; McDonald, 2019). Research has demonstrated that #MeToo and other viral social media movements have raised awareness of sexual assault and led to greater recognition of past unwanted sexual experiences as assault among victims (Jaffe et al., 2021; Kilimnik et al., 2024; Palmer et al., 2021), which may impact disclosing violence and help-seeking behaviors. Disclosure and calls to action might increase due to what Suk and colleagues (2021) call a “network of acknowledgement” that creates a connected community of support; however, this form of activism may also enable or amplify backlash, leaving survivors vulnerable to online abuse and regressive “victim blaming” discourse, which can perpetuate feelings of self-stigma and shame ultimately resulting in further silence or revictimization (Alaggia & Wang, 2020; Gieseler, 2019; Gjika & Marganski, 2020; Warnecke & Kacos, 2023).

Chronologically, these movements have also coincided with the political polarization of sexual violence following the 2016 Presidential Election and appointment of controversial figures to high-profile leadership positions (e.g., Betsy DeVos, Brett Kavanaugh) during the Trump administration. The governmental protections for victims of campus sexual assault that were strengthened between 2011 and 2016 faced significant criticism and were rolled back via the U.S. Department of Education under Betsy DeVos (Yoffe, 2018).

While literature is emerging on how social movements and high-profile events may affect individual perceptions and behaviors, few studies have examined impacts at institutional or campus community levels. Measuring historical and social context is critical for rigorous program evaluation as capturing “history effects” facilitates control of external variables and environmental factors that may influence study outcomes. (Ferguson, 2004; Supino, 2012).

## Current Study

The purpose of this study was to describe sociopolitical events and national discourse (i.e., “history effects”) and their perceived impacts related to sexual violence on college campuses. This paper presents findings from a secondary analysis of qualitative data to answer the research questions, “What issues or events have resulted in greater attention to campus violence and violence prevention efforts from the perspective of college campus personnel?” and “What are the perceived impacts of these issues or events?” Data come from a larger efficacy study collected from key informants working in violence prevention at universities receiving federal funding. This study aims to provide important insights into how the historical context in the United States between 2017 and 2019 may have impacted how campus communities perceive and address sexual violence.

## Methods

### Primary Study

The Multi-College Bystander Efficacy Evaluation (mcBEE) was a CDC-funded evaluation aimed to determine the relative efficacy of bystander programs at multiple college campuses, with the goal of identifying effective core bystander intervention components associated with increases in bystander behaviors and reductions in violence acceptance, victimization, and perpetration (see Bush et al., 2019).

mcBEE used a concurrent and sequential mixed methods triangulation design over multiple years; the methodology has been described extensively elsewhere (Bush et al., 2019; Clear et al., 2020; Coker et al., 2020; Davidov et al., 2020). Briefly, institutions of higher learning in the United States that were public universities or colleges (and thus receiving Title IX funding) and had an undergraduate student body of at least 10,000 were eligible to participate in mcBEE. Data collection occurred in three phases: 1) review of campus websites for information about bystander program offerings; 2) brief key informant web-based surveys with campus personnel with a role in bystander program adoption or implementation and surveys administered to undergraduate students; and 3) in-depth, semi-structured interviews with key informants who completed surveys in Phase 2. The final sample size was 19 college campuses from across the United States.

### Identification and Recruitment

Staff from universities with a role in bystander selection, implementation, or data collection were invited to participate in a web-based survey using research electronic data capture (REDCap) survey software (Harris et al., 2009). At the end of the survey, key informants were invited to participate in a follow-up telephone interview. Staff were identified via 1) website reviews, 2) snowball sampling (e.g., asking existing survey participants to name others who might be interested), and 3) personal communication between study team members and campus personnel (e.g., asking a known colleague or acquaintance at a university to suggest potential eligible staff members to contact).

### Procedures

At the conclusion of the survey, campus personnel were asked if they would be interested in participating in a follow-up interview to expand on their responses. Participants who expressed interest were contacted by a research assistant to schedule a 60-min interview. The investigator (DD) called the participants at the scheduled time and conducted each interview. To begin the call, the researcher read an informed consent script, asked the participant if they had any questions, and requested permission to audio record the interview. The interview followed a semi-structured format wherein key questions about bystander program characteristics, adoption, implementation, and evaluation were asked, but the interview structure provided adequate space for participants to share other relevant information they deemed important. Each participant received a $10 Amazon Gift Card for their participation sent via email immediately after the interview. Audio recordings were saved to a secure location and sent for transcription via Rev.com. Identifying information (e.g., names and locations) was removed to protect the identities of universities and key informants.

### Measures

The semi-structured interview guide used was designed to collect detailed information about existing bystander programs and their components from each participating campus. Questions focused on 1) how programs were selected 2) resources and capacity required for program delivery, and 3) strengths and challenges related to implementation. While data from key informants informed mcBEE's overarching cost and efficacy analyses, they simultaneously provided robust contextual information about how programs are adopted and delivered on college campuses. The final question in the interview guide asked key informants about the extent to which they and their campus community (administration, students, etc.) perceive violence to be a problem on their campus. To identify history effects and potential impact on bystander or violence prevention programming, participants were also asked to share any perceived campus climate issues or recent events (e.g., new policy implementation, news coverage of high-profile sexual assault cases that may have received extensive news coverage) that may have heightened or drawn attention to a focus on sexual assault or violence/violence prevention.

As often occurs in qualitative research, we uncovered noteworthy and unexpected answers to our question about history effects. Thus, in line with the iterative and flexible nature of qualitative inquiry, as interviewing progressed, we modified the interview guide to include more direct probes about the types of impacts we learned about from participants. This modification occurred about two-thirds of the way through the interviews. Revised probes typically included phrases such as, “Some people we have spoken with so far have mentioned impacts of [issue/event]. Have you noticed any impacts on your campus or community stemming from [issue/event]?”.

### Data Analysis

Important and unexpected insights emerging from the primary data were explored using retrospective interpretation (Thorne, 1998, Thorne, 2004). Retrospective interpretation is a form of secondary qualitative analysis in which new research questions emerge during the original study.

Following Braun and Clarke’s (2006) six step approach to thematic analysis, we used both directed and open conventional content analysis (Hsieh & Shannon, 2005). Although we had pre-determined ideas of what codes and concepts to include and understood that most of the relevant data for this analysis might come from the interview guide question about history effects (directed approach), we also recognized that pertinent information might emerge at any point during the interview. First, DD began the coding process by reading and open coding all transcripts for relevant events (and their subsequent impacts) that may have elevated attention to issues related to violence and/or prevention. This process resulted in preliminary codes that facilitated subsequent coding. Next, the transcripts were divided into thirds and each coder (DD, CD, DW) read and open coded their assigned transcripts, paying special attention to initial codes developed by DD but also individually taking notes of new emerging concepts. Then, we held a debriefing session to share our coding process and develop a revised coding structure. Having three coders allowed us to arrive at consensus for final codes by having the third coder “break the tie” on any discrepancies in coding; however, all coders were generally in consensus about code names and structure. Each coder independently re-coded each assigned transcript according to our revised coding structure and then we worked together to split, collapse, and further revise codes and categories into a hierarchical structure resulting in five key areas (themes). Each theme was paired with illustrative quotes and their related impacts were characterized as either positive or negative.

Trustworthiness and methodological rigor were addressed in several ways. First, we had multiple methods of triangulation. Our original dataset included source and method triangulation (i.e., website review, survey, and interview data) and having three independent coders and multiple debriefing sessions increased both the accuracy and reliability of findings. The first author also read and coded all transcripts, resulting in double coding of all data. A common limitation of secondary data analysis occurs when the original data were collected by people other than those conducting the secondary study; however, the first author collected all interview data and is intimately familiar with the nature and context of each conversation. Further, in this paper we provide a rich description of our methods, including transparency around iterative revisions to the interview process during the study course.

## Results

Of the 76 individuals completing web-based surveys, 74 expressed interest and provided availability for a follow-up telephone interview. Of these, 68 participated in a telephone interview. In two instances, two participants attended and shared a singular interview. Five of the participants completed a second interview approximately one year after their first interview. Typically, one or two (and occasionally three) key informants were interviewed from each campus. Participants worked in the following roles: Title IX Directors, Officers, and Coordinators; Deans of Student Affairs and Student Conduct; Police Officers from Campus Security; Directors and Coordinators from Health Promotion and Wellness units; Directors and Coordinators from Violence Prevention and Advocacy Offices; Health Education Specialists; Representatives from Fraternity and Sorority Life. Interview content included information sharing and discussion related to socio-political and/or other historical events that shifted perspectives of violence and violence prevention on campus or in the community. Analysis resulted in five emergent themes: 1) Changes to Title IX Policies and Regulations; 2) High-Profile Cases of Sexual Assault; 3) the 2016 Presidential Election; 4) The #MeToo and It’s On Us movements, and 5) The Brett Kavanaugh Nomination to the U.S. Supreme Court. A cross-cutting theme related to college campuses as a microcosm for U.S. society also emerged. Below a summary of each theme and event are included, along with illustrative quotes (Table 1) and their subsequent perceived impacts as reported by participants (Table 2).

**Table 1:** Emergent themes paired with descriptions and relevant quotes

Theme and Description	Exemplary Quotes
**Title IX Policies & Regulations.** Obama-era policies surrounding campus sexual assault and guidance from the 2011 Dear Colleague letter resulted in changes to violence prevention efforts, namely provision of additional resources (e.g., staff, funding, offices) and greater prioritization of violence prevention. Uncertainty and confusion occurred after the Department of Education under Betsy DeVos rescinded these without replacement guidance, leaving campus personnel unclear about which processes to follow	*I think just with all the Dear Colleague Letter, the Title IX investigations, all of that has had this issue high on the list of campus administration here. Now with all the potential changes or some changes happening in that realm, it stays on the front burner. (Prevention Director)* *I wouldn't be at all surprised if another two years into this presidential administration those 2001 OCR [Office of Civil Rights] letters…the importance of all of this work suddenly gets withdrawn and no longer important…It's a wild card. (Dean of Students)*
**High-Profile Cases of Sexual Assault.** Allegations of sexual assault or misconduct that garnered widespread local or national attention had cultural and structural impacts. Universities were sometimes “forced to reckon with” gender-based violence issues publicly. This often resulted in instances of student activism followed by more resources (and staff) dedicated to violence prevention. Some participants saw greater support across the university after such events, while others perceived mistrust resulting from how their university responded to allegations	*Each time they [high-profile events] hit, it has really taken over the dialogue around campus and shifted people's focus toward sexual violence and relationship violence and what we might do to change the narrative. (Title IX Coordinator)* *[Administrators] do [take violence prevention seriously] now, because we have been in the news a lot…we have had so much bad press, and so many lawsuits.. It's so annoying to get all these media requests, and a lot of things that are reported in the media are not the whole story, but one positive impact is that our president and our board of trustees are tired of being in the news looking bad, and so they take it seriously. (Violence Prevention Committee Chair)*
**2016 Presidential Election Period.** The time period before and after the 2016 Presidential Election resulted in more dialogue and conversation about gender-based violence. Greater support for survivors, including perceived increases in disclosures and help-seeking behaviors also occurred. Some participants perceived greater or less support for changing norms surrounding violence acceptance, while others discussed a shift in their campus climate that was more openly hostile or unsafe. Several personnel reported more instances of violence, including verbal and sexual harassment, occurring on their campuses, while others suspected decreased rates of reporting sexual assault resulted from the national conversation taking place at the time	*This is a bold claim I can't back up empirically, but if there were big studies of public opinion that would come out in 10 years, and they found, for example, that rewarding someone who had recently been probably accused of sexual assault over and over and over again with the most powerful position in the world, had an impact on reporting rates, or whether or not people felt like they should talk about what's happened to them. If that data eventually came out, I wouldn't be surprised. (Title IX Specialist/Prevention Educator)* *Then I think if we want to talk nationally, the new presidency has brought a lot of violence to campus…Even over the past two years, our support numbers have increased every semester to more and more students utilizing those services. I see that more students are coming forward and feel comfortable doing so, or the number of incidents are increasing. I think it's a little of both. (Dean of Students)*
**#MeToo & It’s On Us Movements.** Viral social movements such as #MeToo and “It’s On Us” normalized the conversation surrounding sexual assault and gender-based violence. This resulted in increased student activism and facilitated prevention programming for educators. In some cases, these movements and resulting discussions led to significant changes, such as more resources, funding, and staff dedicated to violence prevention and policy revisions within campus units that have not historically addressed sexual misconduct. In a few instances, decreases in male engagement in prevention programming was noted as related to #MeToo	*I've ran into no one who hasn't heard of the Me Too movement in some capacity. And so I think it's just given us some additional dialogue as a campus. In terms of programming efforts, slight changes in that we would bring in that as a national conversation and as a starter point for some of our programming pieces. (Director of Violence Prevention)* *I think that the Me Too movement and that campaign Enough is Enough, Time's Up, those things have really been really helpful to shed light on the systemic issues that exist in our society. And then bringing in some of that prevention programming to say, "Let's raise some awareness around these issues and figure out how we can shift culture, change behavior, address behavior, in a way to let folks know that the time is up." To say, "It's time to start changing these cultures. We're not gonna ignore it anymore." (Director of Title IX)*
**Brett Kavanaugh Nomination to U.S. Supreme Court.** The nomination of Brett Kavanaugh to the United States Supreme Court and subsequent televised Senate Judiciary Hearings related to Dr. Christine Blasey Ford’s allegations of sexual assault led to significant impacts, namely among students, including survivors. Participants observed increased dialogue and supportive resources for students. Some participants perceived more disclosures and help-seeking behaviors, while others noted survivors felt re-traumatized or discouraged from reporting due to fears of not being believed or vilified. Several participants	*The Kavanaugh trial was huge. That to me is at the same level as the election, and the Me Too, meaning that it was something that we used and that our students were talking about. How do we digest and sit in some of those tensions that folks were feeling. And so that was something that we did have some conversations on in our respective presentation and things like that. (Assistant Director of Health Promotion)* *Most recently, I think that was just last fall, during the Kavanaugh confirmation hearings, during Dr. Ford's testimony period when she spoke a lot about her experience, and then again I think the media really just took that story and ran in terms of Kavanaugh's approach versus her approach. So we saw again, just a very high number of students that were coming in that were wanting to disclose sexual assault related behavior. Again, that could've been behavior that had just happened, was currently happening, had happened to them previously. (Associate Director of Violence Prevention)*

**Table 2:** Perceived positive and negative impacts of each theme

Theme	Perceived Impacts (Positive)	Perceived Impacts (Negative)
Title IX Policies and Regulations: Obama-era Policies, 2011 Dear Colleague Letter, and Department of Education policy changes under DeVos Administration	More attention and resources dedicated to violence prevention, including bystander programming Violence prevention put on the “front burner” Holistic assessments of campus violence prevention and response efforts	University-level changes seen as simply “checking a box” to be in compliance with federal policies Concerns over new policies not being “survivor-friendly”
High-Profile Cases of Sexual Assault	More dialogue about violence prevention More resources dedicated to violence prevention, including staff	Negative attention on campus in the media/press, both locally and nationally Mistrust in university if allegations perceived as being “covered up” or not taken seriously
2016 Presidential Election Period	More dialogue about sexual assault and gender-based violence More disclosures of sexual assault Increased support for survivors Increased student activism Changing norms surrounding violence acceptance (increasingly positive)	More hostile/less safe campus climate New decreases in reporting sexual assault Increases in violent incidents, including verbal and sexual harassment Changing norms surrounding violence acceptance (increasingly negative)
#MeToo & “It’s On Us” Movements	More attention and resources dedicated to violence prevention, including bystander programming Normalized conversation around sexual assault and gender-based violence More funding for prevention Increased student activism Policy and procedure changes to address sexual misconduct	Decreased male engagement in prevention efforts
Brett Kavanaugh Nomination to U.S. Supreme Court	Increased dialogue More support for survivors Increased service access and help-seeking behavior (i.e., more students seeking services)	Re-traumatization of survivors Not enough resources to address the “flood of students” needing services Students feeling discouraged about disclosing or reporting sexual assault New decreases in reporting sexual assault

### Changes to Title IX Policies and Regulations

Participants discussed how federal regulations issued by the Department of Education, including changes to Title IX policies and recommendations, spurred new violence prevention efforts on their campuses. Key informants described a “flurry of discussion” about Title IX stemming from federal mandates and Obama-era policies after the 2011 Dear Colleague Letter and passage of the Campus SaVE Act in 2013. Some stated that these initiatives facilitated “good momentum” and allowed violence prevention to be put on the “front burner”, resulting in more attention and resources allocated to bystander-based violence prevention. Several key informants perceived the dedication of additional resources was motivated primarily by administrators wanting to avoid paying fines for non-compliance with federal policy. In some cases, new offices (e.g., Crisis Intervention, Institutional Equity, Title IX), positions (e.g., Clery Compliance Officer, Title IX and Diversity Officer, Prevention Specialist), and policies were established, often resulting in hiring new staff dedicated to ensuring trainings and requirements compliance. Some participants firmly believed that campus-level Title IX and programmatic changes occurred only because of federal mandates obligating institutions to provide bystander training. A key informant with an administrative role in the campus health and wellness office shared:

Now, as far as upper administration’s view, what they prioritize or where the attention goes, I would have to say that the only reason that prevention gets any attention, in any significant way now, is probably as a result of the federal mandate...what helps to facilitate those kind of conversations, and that kind of attention, is the federal mandates did not hurt. They certainly shined a spotlight. They didn't necessarily give all the right guidance, but they at least got attention to the work that either had already been occurring for years but wasn't getting enough attention, or respect, or resources, or like I just think that was a shot in the arm to getting people to sort of come around to understanding that this work is not easy, that it needs to be done systematically, that it needs to be done year after year, that it needs to be constantly attended to and intensified, if it's gonna have any impact at all.

Several participants described how their institution was initially reluctant to highlight violence prevention because administrators “don’t want the institution to not seem safe” if reports of sexual assault were to increase. Participants discussed a range of motivations for administrators to prioritize prevention work, ranging from believing it is a priority for the safety of their campus to wanting to complete a checklist for risk management as mandated by federal policy. One participant working with students in Greek Life noted:

There's a constant fight about what gets into orientation and what's important when so many people see it as key. But just the length of the day and the length of orientation already, people don't necessarily want to do an additional thing. I think it's a priority for some and for others it's more of a risk management requirement….It's not organic here at the institution that this is important yet.

Even in instances where prevention efforts were enhanced to “check the box” of federal requirements, many institutions used the new mandates as an opportunity to conduct a holistic assessment of campus violence prevention and response efforts. Participants then described changes to university office structures and bolstered efforts that resulted to ensure compliance with new regulations. Another participant working in their institution’s Title IX office described how increased attention and resources devoted to Title IX initiatives have increased awareness of students’ rights, due process, and reporting of sexual misconduct and assault. They stated, “Within the last year or two, the Title IX office has grown by 300% when every other office is shrinking.” Some participants suggested that higher rates of reporting have been observed following these changes to Title IX processes and regulations, suggesting college students are better able to seek and receive help. 

Further, throughout discussions about how mandates and federal policies shifted attention to campus violence prevention efforts during 2014 – 2016, some described that the recent momentum was stalled or even reversed by the “constant flux of policy changes and mandates” that occurred after the 2016 Election when Betsy DeVos was appointed as the Secretary of the Department of Education. The participant that noted their Title IX office grew by 300% after the federal mandates added:

It's been fascinating and then deflating to watch this cycle where the [2014] Dear Colleague letter happened, and then all of a sudden when it feels like nobody has been paying attention, now everybody's paying attention, and there's all of this forward momentum, and then the backlash happens...So you feel the sense of a forward momentum, and it's mostly positive, and then the reverse happens.

Several participants described apprehension and concern about the anticipated changes at the Department of Education related to prevention work on college campuses. Specifically, there was consensus that significant changes were forthcoming, but there had been little direction provided to campuses about when those changes would occur or what they would be. During their second interview in 2019, one participant stated that the DeVos administration revoked the Obama administration’s guidance on sexual violence prevention policies without replacing it with substantive direction:

You wonder when these policy changes become effective, how much are we going to have to adjust?...Definitely their strategy of repealing all of the Obama-era guidance without replacing it with anything is also a problem. So for the last couple of years, it's just been like nobody knows what the expectations are, at least as far as the Department of Education is concerned. it's a bleak picture, but that's where we are.

Another participant working in the women’s center on campus echoed this concern:

They were talking about the ways and some of the things that were happening at the national level, were going to be trickling down to us, but at the time they didn't know exactly what that meant. They knew that there were going to be implications for our campus in the way that we handled things, but at the time they didn't know exactly what those things were going to be. So that was the last that I heard….They did a PowerPoint and they had how some of the legislation was changing and that they thought that the changes that were coming were not going to be positive changes, but they, I guess, positive in favor of victims rights….It was the implication, but they didn't have specifics yet, because everything wasn't in stone, in the government yet. So I don't know, I know that we are feeling those effects. I know that Title IX is feeling those effects, but exactly how they're feeling it and what those things are, I'm not sure….Yeah. They don't sound very survivor friendly.

### High-Profile Cases of Sexual Assault

When asked whether there were events they could recall that have elevated attention to violence, numerous participants referenced high-profile cases of sexual assault or other types of sexual misconduct, including those that received national attention. Frequently mentioned events included those involving prominent athletes, widespread media coverage of stories focused on universities’ responses to high-profile cases, and public lawsuits against alleged perpetrators of violence. Some participants spoke about internal events that may not have garnered national attention but had serious impacts locally and on their campus communities. Examples include a case where a faculty member was murdered by an abusive partner, an accusation of sexual harassment by a faculty member that was allegedly covered up and sealed, and an instance of serial sexual violence perpetrators on one campus. Hazing, harassment, and assaults occurring among students affiliated with Greek Life were also noted as bringing increased awareness and attention to these issues. Participants also discussed how high-profile cases of sexual violence in the news at other campuses or at peer institutions affected their own campuses.

Participants shared two perceived positive outcomes or impacts of these events: increased dialogue and the provision of more resources. One compliance officer said, “Each time they [high-profile events] hit, it has really taken over the dialogue around campus and shifted people's focus toward sexual violence and relationship violence and what we might do to change the narrative.” A Title IX coordinator described the way their university was handling a series of very public cases of sexual assault and misconduct, sharing:

It was a case where there was a lot of publicity around it and I think for probably the first time the community started to see the commitment from institutional leaders who were giving press conferences and speaking about different elements, and the university’s handling of the case, and the fact that individuals were held accountable. Actions were taken. Complaints were taken seriously. It was maybe the first time that people had seen how the university, in a very public way, responds and handles something. Where I think prior to that because the university doesn't generally talk about complaints there was sort of this perpetuating myth that nothing had changed and everything was business as usual. So I feel like there's maybe been a bit of a shift and that we're starting to communicate more and get the word out more about our efforts and what has changed over time.

One key informant in a leadership position in a Title IX office was asked if they could recall any events that have shifted attention onto violence prevention, and replied, “Yes. And it's why I have a job. And it's horrible.” They continued to describe several instances of alleged sexual assault and intimate partner violence perpetrated by student athletes and explained how the university’s response garnered widespread media attention. Thus, in addition to federal mandates and investigations (Theme 1) spurring the creation of new offices and positions dedicated to violence prevention on campus, so did highly publicized cases of sexual assault and violence (Theme 2). Another Title IX official said:

I think there was a lot of good work on the campus being done before that [one big case that ‘blew up’ in the media]. But it was very siloed and not as coordinated. There weren't as many resources being dedicated to it. The after was that you had a significant influx of resources being dedicated to these issues. A lot of attention on and off-campus. A lot of scrutiny. With that can come some good things as well as being difficult.

A director working in a prevention office added:

With the national scandal here on campus, and all of the federal and national focus, there has been a dramatic increase in both prevention and response on campus. Both of them were beefed up quite a bit…. And unfortunately it took a national scandal, but we got a lot more resources.

Overall, interviewees described various instances of these high-profile cases having a widespread cultural and structural impact institutionally, wherein more resources, support, and communication about gender-based or sexual violence issues emerged. For some campuses, especially those where a case resulted in significant negative attention or press on the university, participants shared how sometimes the event resulted in greater introspection and conversations among administrators, where they were “forced to reckon with” gender-based violence issues publicly. Sometimes university responses occurred after student rallies, protests, and other displays of student activism (e.g., videos shared across campus and via social media). In some cases, because of public pressure and negative attention on the university, advocacy and violence prevention centers and offices received more funding and support or were established for the first time. There was consensus that high-profile events resulted in the hiring of Title IX Coordinators and dedicated full-time staff focused on violence prevention than campuses previously had. Several participants reported that their universities developed more comprehensive sexual misconduct policies and made significant changes to the university organizational structure, including the composition of university divisions (e.g., student life, student misconduct, etc.) with a special emphasis on student safety and support. Conversations and collaboration across campuses and between different divisions—that previously worked in isolation or did siloed work—would also occur because of high-profile events.

Participants also spoke about university administration taking accusations of sexual violence or misconduct more seriously than before the significant event occurred. One participant who chaired a violence prevention committee on campus stated:

It's [high-profile event] been a real change...It used to be, if we get a complaint about a high-level administrator, or a high-level faculty member, it was like, “Oh no, how are we gonna prevent this from getting out?" I didn't get the impression that it was like, "Let's pretend it's not happening," but it was, "Let's try and keep this as discreet as possible when we address it.” Now it's like, "Okay, can we fire them yet? Let's fire them. Hurry up and finish your investigation so we can fire them.”

Participants reflected on how they believed students and campus communities perceived the impacts of the high-profile events they discussed. Participants spoke about how the university might receive support and praise when they spoke out against allegations of sexual assault and misconduct; however, mistrust and doubt also resulted if there were perceptions that the university was not being proactive or taking allegations seriously. One participant said:

[The] university wants to remain silent. So unfortunately, the university, the only response that happens is maybe a poster campaign will go out, or maybe suddenly we'll see a surge in requests for education. But our university is not known for making statements of support…in fact I would say high-level cases at the university involving sexual assault have been very well covered-up by the institution. Title IX will get involved, and absolutely will not involve us.

Taken together, there was consensus that although high-profile cases of sexual violence were difficult, and oftentimes triggering to students—and especially survivors—these events and subsequent widespread attention often resulted in important dialogue surrounding campus sexual assault as well as more students seeking services. Personnel working in violence prevention on campus mentioned using the emerging conversation surrounding sexual violence to advocate for more resources to ensure compliance with federal policies. For example, a Title IX Coordinator said:

And you have to jump on that, right? Title IX, it's still hot, unfortunately. It's still hot in the national news, there's still headlines almost every day in this area. I've tried to partner, and help...What can I do to help you get what you guys need? Just because it doesn't reside in my office, doesn't it mean I can't make that pitch. And hey, this is what we need; not only to be in compliance, but here are the resources we need for our students and staff.

Further, one prevention educator used high-profile events as an attention grabber or teaching moment during violence prevention programming, sharing that the sexual assault case “…was at a high school here in [state]. So I will say that using that case is often an especially effective teaching example for [university name] students because it feels so real to them. Like it happened right here.”

### 2016 Presidential Election Period

Nearly one-third of participants discussed impacts of the 2016 Presidential Election on their campuses. Perceived impacts ranged from more conversations about sexual assault, higher rates of disclosures, and even increases in harassment and violence on campus. Several specifically mentioned the release of the “Access Hollywood Tape”—a video published by the Washington Post on October 7, 2016 in which then-presidential candidate Donald Trump was discussing attempts to seduce a married woman with TV host Billy Bush. The video depicts Donald Trump saying, “I don't even wait. And when you're a star, they let you do it. You can do anything… Grab 'em by the pussy. You can do anything.” (Bullock, 2016) There was consensus that the political discourse and national discussion surrounding the video, including Donald Trump’s subsequent apology where he equated his words with “locker room talk”, created more conversation about gender-based violence and sexual assault, especially among students. Some participants perceived that this resulted in new decreases in reporting, while others believed disclosures and reports of sexual violence were increasing. A key informant from an office for sexual misconduct said:

The national conversation has also lent itself to allow students to speak about experiences that they've had. So I'm not quite sure if I would say the behaviors themselves are happening at a higher rate, or a substantially higher rate, more so than just students are now feeling okay talking to someone at the university about their experience.

Several participants, including a sexual assault prevention coordinator, perceived a shift toward a more unsafe climate for students on campus, including instances of verbal harassment:

I think that the climate has shifted quite a bit this last year, since last November. There's been a real increase in verbal and harassment behavior towards students of color, students from different religions, and international students, and LGTBQ students. And I don't know if that's gonna calm down, but ever since the November elections, I've seen a generalized increase in that kind of behavior, which creates a very unsafe feeling or climate, I think, for students.

Another coordinator from a violence prevention office shared:

I think that also, certainly with the current national White House administration, we have seen a rise in street and sexual harassment, as it relates to gender. We have had multiple assaults that involved literally grabbing female students by the pussy…And in some pretty violent ways as well, but that is definitely a distinct and new thing that we are seeing, right?

Participants recognized the impact the media coverage and conversations related to sexual assault at the local and national levels may have on survivors on campus. Some discussed enhanced efforts on campus to provide support for those students:

We’re gonna have to really make sure that we're taking care of our survivor students, 'cause they're reading this in the paper, people are gonna be discussing it in class, and if you've already been traumatized, it's gonna be really difficult to have that happening in constant conversations. So, we just really have to boost up our support to those students, strengthen those students up.

In addition to bolstered resources, several participants noticed a shift in norms surrounding violence acceptance. Perceptions of how norms changed varied; some felt this period led to greater attitudes of violence acceptance, while others believed standing up against violence was becoming normalized. A director from a violence prevention office said,

Another thing I've noticed, kind of I think tied to that, is students being a lot less tolerant to people being, for lack of a better word, problematic or harmful. One thing that I remember—the year that we were still doing [bystander program]—our last session was after the presidential elections. Someone even said, "To play devil's advocate, what about this?" And one student in that session just turned around and said, "You're not allowed to say that anymore." Shut it down.

### #MeToo & It’s On Us Campaigns

Soon after the 2016 Presidential Election, the #MeToo movement began in 2017 when the Twitter hashtag, founded by Tarana Burke, went viral. Around the same time, the “It’s On Us” Campaign, which started in 2014 as an Obama-Biden administration initiative, grew into a large non-profit focused on campus sexual assault. The “It’s On Us” slogan and messaging was adopted by numerous college campuses to market violence prevention programs and services. In fact, multiple campuses participating in mcBEE considered “It’s On Us” as one of their bystander-based prevention strategies to address Title IX/Campus SaVE requirements. Both viral campaigns were discussed by participants as normalizing conversations surrounding violence prevention. Those personnel responsible for implementing violence prevention programming also discussed that being able to reference national movements helped introduce these issues to students. One violence prevention and survivor advocate said:

Tarana Burke and the #MeToo movement, and kind of the adoption by white women with privilege, has helped kind of normalize some of these conversations, and made it easier for people to identify what's happened to them. I think that that's super important.

Multiple participants discussed how these movements and the national focus on violence on college campuses led their administrators to devote additional funding and resources to sexual assault prevention. A sexual violence prevention educator stated:

I'm pretty sure that many of the programs that are now happening and the funding that is available for Title IX on our campus is because Joe Biden specifically called [School] and was like "Hey you're one of the universities that's really fucking this up.” That call jump started and greatly increased the sort of funding and attention that is given on campus to rape and sexual assault.

Students also requested more resources and stressed the need for campus-wide dialogue about #MeToo (e.g., “If it was 2016, the year that everything started, we were getting a lot of feedback from particularly students that they wanted to have some kind of a campus wide dialogue about what they were seeing.” (Title IX/Clery Coordinator). Of note, Tarana Burke was invited to speak at several campuses involved in mcBEE, according to those interviewed. One participant shared how they harnessed #MeToo and high-profile cases of sexual assault in the media to offer spaces for dialogue and programming:

This was around the time of Harvey Weinstein and Matt Lauer and all these high-profile cases. So our office actually sponsored a #MeToo week, so we provided a forum for people to host programs to talk about various aspects of sexual violence, of sexual harassment in the workplace, in academia. We did an aspect of international students and harassment abroad and had community agencies come in and do programs. We had faculty do some lectures, and then students put on programs as well as our office hosted some. So it was really great, and that's continued annually now. So that has certainly been something that people are very engaged with.

Some participants reported that viral movements shed light on systemic issues that exist within society and led to campuses being “activated” around violence prevention, especially in units that had not previously developed policies and procedures for sexual misconduct. This led to policy revisions and more resources provided in graduate programs, medical schools, and within academia more generally. A Title IX administrator provided an example:

I think we have seen an increase in conversations, particularly at our med school because there is this MeToo Movement right now within the medical profession. And so I think this overall climate of learner mistreatment within med schools is really being highlighted right now. for us where we're seeing it [the influence of #MeToo] the most is within our med school….[It has] had a huge impact on at least just awareness and conversations within our School of Medicine.

On the other hand, a few participants discussed how the national conversation from viral movements like #MeToo and “It’s on Us” have coincided with decreased male engagement in violence prevention programming. They discussed how some male students felt targeted or “called out” by these conversations. Several interviewees mentioned that the typical student engaged in campus violence prevention is already aligned with active bystanding and anti-violence. They acknowledged the need to reach and engage those who are not yet “bought in” to bystander-based prevention strategies, including men that might feel targeted by such topics. One director of violence prevention in charge of bystander programming said:

Male students' engagement with our programs has decreased every year…I believe that that is a direct response to the national attention on Me Too and the conversations on things like Not All Men, and this feeling that men are being targeted. How does that still impact engaging them into this work? That duality of doing prevention efforts, where we are really trying to target the students that need to be a part of this the most, and also recognize that there needs to be a space for survivors to feel heard. And the reality is most times they're being harmed by cis men.

### Brett Kavanaugh Nomination to U.S. Supreme Court

In 2018, Judge Brett Kavanaugh was nominated to the U.S. Supreme Court by President Trump to fill Justice Kennedy’s position upon his retirement. During the nomination and confirmation process, Dr. Christine Blasey Ford privately, and then publicly, alleged Kavanaugh sexually assaulted her in 1982 when they were in high school. Dr. Ford’s subsequent testimony before the Senate Judiciary Committee was televised and received nationwide attention (McGinley, 2019). The hearing and eventual confirmation of Brett Kavanaugh to the U.S. Supreme Court by a senate vote of 50–48 undoubtedly made waves at college campus levels, according to many participants interviewed. After Dr. Ford received criticism for waiting too long to come forward with her accusations, sexual assault survivors on social media began disclosing their experiences using the hashtag #WhyIDidntReport. Similarly, multiple participants in this study shared how more survivors on their campus were seeking services as a direct result of watching Dr. Ford’s testimony or its news coverage. When reflecting on conversations with students, one administrator in a sexual violence prevention and support office said:

[Students would say] “I was watching the news and talking about this with my friend and now I feel like I finally feel comfortable talking to someone about this." Or, "Someone had this on in the background and the news was talking about the nature of the allegations or something, and now I feel like I need to talk to someone." So it was a very, very, an explicit line of, because I had heard this or seen this, via the Kavanaugh confirmation hearing, then I now want to seek out your office for services.

Participants discussed how the hearing and subsequent disparagement of victims re-traumatized survivors. For others, it led to recognition that something they had experienced in the past may have been considered sexual assault. In some cases, this resulted in more formal reports of sexual assault, as one Title IX official stated:

And so our advocacy centers received kind of this flooding of students just coming in and being re-traumatized or realizing for the first time what they experienced might have actually been some form of sexual assault or sexual harassment. And so we've seen a lot of that and we're still kind of navigating through the aftermath of the Kavanaugh hearing.

While many participants perceived increased reporting, others discussed how the Kavanaugh hearing had the opposite effect and may have even discouraged reporting by some students. One violence prevention specialist reflected on students’ perceptions related to reporting:

[Students would say] “Well, I can't report now. There's no way I could report this athlete now that I know that this is how the media or social media is going to respond, or my fellow students are going to respond." Or "Look what happened with Kavanaugh. Even if I report nothing will ever happen and I have to stay silent."

This participant continued, “We are anecdotally hearing from survivors that they do not feel safe to move forward with reporting if that is what they wanted. Then deciding that they just can't because of climate on our campus and nationally.” They went on to describe how the university’s student government association asked that more mental health staff be hired specifically because of increased rates of depression, anxiety and suicidality. An advocate working in a violence prevention office shared how the widespread attention on the hearing led to the university putting out a statement of support for survivors, underscoring the gravity of the hearing’s impact:

Folks were definitely talking about Kavanaugh, and we saw our I think workshop participants impacted by that. Definitely our peer educators were really struggling with doing the work for a while. As pro staff, we definitely talked about it, and our University Health Services actually ended up…putting out a statement of survivor support on the homepage, so that was exciting.

### Cross-Cutting Theme: Campus Problems are Societal Problems and Societal Problems are Campus Problems

Participants shared that all five socio-political issues and events described here spurred heightened awareness and increased dialogue surrounding sexual violence on their campuses. In some cases, they perceived that increased (and sometimes decreased) reporting and more resource-seeking among students resulted. Some campus personnel described the provision of additional resources dedicated to violence prevention after significant events. In sum, small and large-scale societal issues permeate college campuses and communities in myriad ways. Further, these data highlight the various ways in which college campuses serve as a microcosm for U.S. society (Jones, 1990) and how university-level events can infiltrate and sometimes shift public and national discourse. One participant aptly stated, “So much of the way we understand our local world has just become nationalized…But I do hear students, staff, and faculty alike make reference to national events on a big scale.” While these evolving conversations are paramount for mitigating violence, a prevention educator working within a Title IX office cautioned against exclusively concentrating on sexual assault as a concern limited to colleges or universities. He emphasized that true violence prevention at the societal level demands comprehensive strategies that engage younger age groups and foster transformative changes from an early age:

I think what frustrates me in large part about this whole realm is that our national dialogue is focused so much on the issue of campus sexual violence. For me, it's the issue of sexual violence in our society and our culture. That by focusing only on campuses we're really narrowing it in an unhelpful way sometimes. We need to be educating people about healthy sexual relationships and what consent looks like and how to talk about sex much earlier than college…We need to be focusing on doing this in middle school and high school because by the time they get here, there's a lot of behaviors and understandings that are already in place. Then we are holding them accountable to standards that we haven't actually been teaching folks when they are growing up and when it could really be more effective.

## Discussion

The current secondary qualitative analysis was unique in its direct examination of the perceived impacts of national and local socio-political context on dialogue and action surrounding violence prevention and response on college campuses. Data for the present study were collected between 2017 and 2019, an important socio-political time in the United States and globally. The U.S. presidential election and subsequent changes in administration, important movements and widespread coverage of sexual assault cases, and changes in Title IX legislation and related regulations, for example, led to shifts in the socio-political climate and elevated public discourse surrounding sexual violence in the media, online (i.e., social media), and in communities. Consistently, and as data obtained in the present study demonstrate, these shifts were also observed and experienced on college campuses and significantly impacted student and staff behavior and overall campus practices, policies, and implementation of violence prevention programming.

Pre-2017, many U.S. college campuses, particularly those receiving federal funding, were responding to the “Dear Colleague Letter” and subsequent documentation and materials (U.S. Department of Education, 2014) as well as high-profile coverage of cases of college sexual assault. These documents, policies, and events, along with amendments to the Clery Act (e.g., Violence Against Women Act, Campus Sexual Violence Elimination Act) expanded awareness and provided revised guidance and examples of sexual violence definitions, policies, practices, procedures, and programming with the intent to improve and grow efforts targeting and preventing sexual violence on college campuses. (Rape, Abuse & Incest National Network, 2023). Still, responses to these government recommendations varied, subsequently influencing interpretations of sexual assault risk and protective factors and the best practices for prevention and response strategies to address campus sexual violence (e.g., Amar et al., 2014; Koss et al., 2014; Moylan & Javorka, 2020; Pappas, 2016). Interpretations and responses by college campuses were further complicated during the 2017 change in U.S. President and Secretary of Education leadership, during which the “Dear Colleague Letter” and related documentation were formally rescinded and replaced with new guidance with limited recommendations and/or directives for implementation. Simultaneously, public discourse around sexual and gender-based violence has grown, largely related to the feminist #MeToo and #TimesUp movements, as well as highly publicized cases of sexual assault in the media (e.g., Ford-Kavanaugh sexual assault hearings) and criticisms of college and university responses to reports and rates of sexual violence. As such, understanding historical effects and lived experiences within this socio-political context was pivotal.

Overall, findings suggest the powerful and salient positive and negative impacts of public discourse on sexual violence, socio-political context, and federal policy (especially related to Title IX) on procedures, policies, and preventive programming implemented by colleges and universities and staff and student behavior. Regarding policy, key informants in the present study demonstrated attentiveness to rules and regulations related to federal mandates/policies and concern for compliance to avoid fines and other negative consequences. Further, participants' responses demonstrated that campus prioritization of violence prevention, subsequent resource allocation, and rates of reports and help-seeking behaviors among college students were impacted by changes to and enforcement of federal policies. Local and national events also resulted in increased public discourse, mass movements, media coverage, and critical attentiveness to the responsivity of college and university settings to socio-political context. Results highlight the need for continued dialogue and increased support and resource allocation for students, especially survivors, to prepare for potential increases in reports, help-seeking, and resource needs during times of significant public discourse, controversy, and political shifts at the local level and beyond. Findings also suggest changing norms on college campuses and among students to normalize conversation around sexual violence and its prevention. This has also been consistently reflected in growth in funding and attention to sexual violence intervention and prevention at governmental levels over the past decade. However, as the 2016 election demonstrated, administrative shifts due to the democratic process, particularly in the United States, can have dramatic and sometimes confusing impacts on universities, their attempts to provide resources and supports to their students, and subsequently on students and survivors themselves.

From an epidemiological perspective, the findings reported here highlight the importance of measuring historical effects during efficacy evaluations. Capturing changes in social and political context is critical for identifying threats to external validity and potential mediators and moderators of program efficacy. Particularly in the context of observational and quasi-experimental studies, researchers should incorporate qualitative and mixed methods approaches such as direct inquiry, interviews, and surveys to capture exposure to other environmental influences to identify contextual factors and experiences that may affect outcome measures (Sacket & Mullen, 1993).

This study is not without limitations. While our sample size was large enough to reach data saturation for each of the five reported themes, other important categories of information emerged that warrant further exploration. For example, lack of support for violence prevention programming among students and faculty was mentioned during interviews. Future studies should explore barriers and facilitators to engagement for violence prevention, especially among high-risk subgroups like cis men, athletes, and students in Greek Life, whose buy-in is critical for successful bystander action and reductions in violence perpetration (Wiersma-Mosley et al., 2017). In this same vein, it is crucial to take college administrators’ perspectives into account. Several participants noted that school administrators sometimes prioritized the school’s reputation over violence prevention and response, worrying that investments in violence prevention might signal that their campus was unsafe. However, some personnel observed campus leadership moving from wanting to “sweep things under the rug” to taking swift action in the form of making public statements and committing to thorough investigation of misconduct cases. Highlighting how programs contribute to campus safety may be important for securing administrators’ buy-in and investment in violence prevention.

Finally, it is important to note that these data represent the subjective experiences and perspectives of those interviewed. Their descriptions of noteworthy events and resulting campus-level impacts reflect their own perceptions and interpretations. While our sample was diverse in the types of personnel recruited for interviews (e.g., Title IX staff, campus police, prevention educators), data triangulation could be enhanced by exploring the experiences and perspectives of administrators, students, faculty, and other campus staff not captured in this analysis. Finally, a common limitation of secondary analysis of qualitative data is that existing data are collected for reasons other than exploring the current phenomenon of interest. This limitation is largely mitigated in the current study because our interview guide specifically contained a question related to the topic of this paper and the first author was the sole interviewer and primary analyst of all qualitative data.

## Conclusion

The present study has demonstrated that college and university settings, including the communities that exist within them (i.e., students, staff) operate within as well as impact socio-political context. In other words, campus-level problems related to sexual violence (e.g., high prevalence, suboptimal prevention and intervention programming and resource allocation) are pivotal societal public health problems while societal problems regarding response and prevention of sexual violence (e.g., policies, regulations) are campus problems, also impacting programming, resource allocation, student and staff behavior, and more. While dialogue is changing around these issues, and public awareness is building vocabulary and important changes to guidance and recommendations for response and prevention, this study demonstrates that we are far from resolving the societal sexual violence crisis experienced by our young adults. As one participant eloquently noted in the present study, the problem is not exclusively sexual violence on campuses, but “sexual violence in our culture.” Sexual assault risk and prevalence data support this idea, as even though college-aged women (i.e., 18 – 24) have the highest rates of sexual victimization, non-college attending women in this age group are 20% more likely to be a victim of rape or sexual assault compared to those who are college students (Sinozich & Langton, 2014). While significant attention and resources have been dedicated to combating sexual violence on college campuses, college students are not a monolithic group representing all at-risk individuals. To promote positive, bidirectional effects, future directions should consider novel strategies for leveraging media, socio-political context, and national and local events in efforts to promote both campus- and larger societal and systems-level climate change surrounding violence prevention. Additionally, it is important to critically examine other mediating and/or moderating factors that impact how individuals, groups on college campuses, and society at large engage in discourse surrounding sexual violence and respond to events in addition to impacts on preventive and responsive actions, and subsequently, rates of sexual violence.

## Data Availability

The de-identified data that support the findings of this study are available upon reasonable request from the corresponding author, [DD]. The data are not publicly available due to privacy and ethical restrictions (i.e., containing information that could compromise the privacy of research participants.
